# Posttrial Withdrawal Ethics in the Healthy Ageing Ecosystem for People With Dementia (HAAL) Study

**DOI:** 10.2196/93636

**Published:** 2026-05-08

**Authors:** Praisewin Johny, Anish KR

**Affiliations:** 1 Rajagiri College of Social Sciences (Autonomous) Cochin, Kerala India

**Keywords:** older people, dementia, dashboard, technology for older adults, digital health, eHealth, innovation in health care

## Letter to the Editor

This letter addresses the protocol by Amabili et al [1] for the Healthy Ageing Ecosystem for People with Dementia (HAAL) study. While the rigorous user-centered design is commendable, an ethical concern about posttrial responsibilities warrants discussion in assistive technology research.

The protocol describes a 3-month deployment of an integrated smart home ecosystem, followed by system removal at the final evaluation [1]. Participants received training, adapted their care routines around the technology, and showed reduced stress in preliminary findings. However, the protocol contains no explicit provisions for posttrial access or management of dependency effects when beneficial interventions are withdrawn.

This raises a critical question: What are researchers’ responsibilities to participants who integrate assistive technologies into daily life, particularly vulnerable older adults with progressive cognitive decline and their already-burdened caregivers?

International research ethics frameworks recognize that responsibilities extend beyond intervention periods. The Declaration of Helsinki requires planning for continued access to beneficial interventions [2]. Clinical trial withdrawal studies document that stopping beneficial interventions causes distress for participants and creates tension between beneficence and research constraints [3]. When assistive technologies become integrated into daily care routines and are then removed, loss of perceived independence or safety constitutes an ethical problem [4].

For people with dementia and their caregivers, this concern carries particular weight. Caregivers may restructure routines around monitoring alerts and experience genuine stress relief. Removing supports after 3 months risks reversing benefits and creating new harms: increased anxiety, perceived safety loss, and care pattern disruption.

Assistive technology feasibility studies should adopt posttrial continuity and transition protocols including (1) explicit disclosure about posttrial plans in consent documents; (2) gradual tapering rather than abrupt removal; (3) transition support periods with counseling and alternative resources; (4) continuation pathways where feasible; and (5) systematic assessment of withdrawal impacts, reported transparently.

This critique does not suggest the HAAL study was unethical; feasibility pilots routinely involve temporary deployment and participants were informed [1]. Rather, as our field matures, frameworks bridging clinical research ethics (emphasizing posttrial access) with technology research (treating devices as temporary interventions) must be developed [5].

The HAAL protocol represents important innovation. Building on this foundation, our community should address the ethical gap between validating assistive technologies and responsibly managing their withdrawal from participants’ lives.

## Abbreviations

HAAL: Healthy Ageing Ecosystem for People with Dementia
